# Protocol for an individual patient data meta‐analysis on blood pressure targets after cardiac arrest

**DOI:** 10.1111/aas.14090

**Published:** 2022-06-09

**Authors:** Markus B. Skrifvars, Koen Ameloot, Johannes Grand, Matti Reinikainen, Johanna Hästbacka, Ville Niemelä, Christian Hassager, Jesper Kjaergaard, Anders Åneman, Marjaana Tiainen, Niklas Nielsen, Susann Ullen, Josef Dankiewicz, Markus Harboe Olsen, Caroline Kamp Jørgensen, Manoj Saxena, Janus C. Jakobsen

**Affiliations:** ^1^ Department of Emergency Care and Services Helsinki University Hospital and University of Helsinki Helsinki Finland; ^2^ Department of Cardiology Ziekenhuis Oost‐Limburg Genk Belgium; ^3^ Department of Cardiology University Hospitals Leuven Leuven Belgium; ^4^ Faculty of Medicine and Life Sciences University Hasselt Diepenbeek Belgium; ^5^ Department of Cardiology Copenhagen University Hospital – Rigshospitalet Copenhagen Denmark; ^6^ Department of Intensive Care Kuopio University Hospital and University of Eastern Finland Kuopio Finland; ^7^ Department of Anesthesiology, Intensive Care and Pain Medicine Helsinki University Hospital and University of Helsinki Helsinki Finland; ^8^ Intensive Care Unit Liverpool Hospital, South Western Sydney Local Health District Sydney Australia; ^9^ University of New South Wales Sydney Australia; ^10^ Faculty of Medicine and Health Sciences Macquarie University Sydney Australia; ^11^ Department of Neurology Helsinki University Hospital and University of Helsinki Helsinki Finland; ^12^ Department of Clinical Sciences Lund University Lund Sweden; ^13^ Anaesthesia and Intensive Care Helsingborg Hospital Lund Sweden; ^14^ Skåne University Hospital Clinical Studies Sweden – Forum South Lund Sweden; ^15^ Department of Clinical Sciences, Lund, Section of Cardiology Skåne University Hospital Lund, Lund University and Clinical Studies Lund Sweden; ^16^ Copenhagen Trial Unit, Centre for Clinical Intervention Research Copenhagen University Hospital – Rigshospitalet Copenhagen Denmark; ^17^ Department of Neuroanaesthesiology, The Neuroscience Centre Copenhagen University Hospital – Rigshospitalet Copenhagen Denmark; ^18^ South Western Clinical School University of New South Wales Sydney Australia; ^19^ Critical Care Division, the George Institute for Global Health University of New South Wales Sydney Australia; ^20^ Department of Regional Health Research, Faculty of Health Sciences University of Southern Denmark Odense Denmark

**Keywords:** cardiac arrest, cardiopulmonary resuscitation, mean arterial blood pressure

## Abstract

**Background:**

Hypotension is common after cardiac arrest (CA), and current guidelines recommend using vasopressors to target mean arterial blood pressure (MAP) higher than 65 mmHg. Pilot trials have compared higher and lower MAP targets. We will review the evidence on whether higher MAP improves outcome after cardiac arrest.

**Methods:**

This systematic review and meta‐analysis will be conducted based on a systematic search of relevant major medical databases from their inception onwards, including MEDLINE, Embase and the Cochrane Central Register of Controlled Trials (CENTRAL), as well as clinical trial registries.

We will identify randomised controlled trials published in the English language that compare targeting a MAP higher than 65–70 mmHg in CA patients using vasopressors, inotropes and intravenous fluids. The data extraction will be performed separately by two authors (a third author will be involved in case of disagreement), followed by a bias assessment with the Cochrane Risk of Bias tool using an eight‐step procedure for assessing if thresholds for clinical significance are crossed. The outcomes will be all‐cause mortality, functional long‐term outcomes and serious adverse events. We will contact the authors of the identified trials to request individual anonymised patient data to enable individual patient data meta‐analysis, aggregate data meta‐analyses, trial sequential analyses and multivariable regression, controlling for baseline characteristics. The certainty of the evidence will be assessed by the Grading of Recommendations, Assessment, Development and Evaluation (GRADE) system. We will register this systematic review with Prospero and aim to redo it when larger trials are published in the near future.

**Conclusions:**

This protocol defines the performance of a systematic review on whether a higher MAP after cardiac arrest improves patient outcome. Repeating this systematic review including more data likely will allow for more certainty regarding the effect of the intervention and possible sub‐groups differences.

## 1 INTRODUCTION

Most cardiac arrest (CA) patients die due to hypoxic brain injury that develops over the first 48–72 h in the intensive care unit (ICU).^1^, ^2^, ^3^ Pilot studies have suggested alleviation of brain injury by targeting a higher mean arterial pressure after CA.^4^, ^5^, ^6^ Increasing MAP by administering low doses of vasopressors is possible after CA; noradrenaline is the usual first‐line vasopressor.^7^, ^8^, ^9^ Vasopressors may have adverse effects, including cardiac arrhythmias and increased myocardial oxygen consumption that can result in ischaemia and afterload elevation.^10^ Severe side effects include re‐arrest and/or the use of mechanical circulatory support.^11^, ^12^ If more intravenous fluid is used to achieve a higher MAP, possible adverse effects could be pulmonary oedema, hypoxia and prolonged mechanical ventilation. As early deaths (1–3 days) after cardiac arrest are commonly due to untreatable circulatory shock or multiple organ failure, we will include ICU and hospital mortality as outcomes to identify severe side effects related to cardiac failure.^10^ Some studies have also investigated whether increasing MAP alleviates brain injury by studying brain injury biomarker levels; we will include these if the reported results enable pooled analysis.^5^, ^6^, ^13^

No conclusive data on the optimal MAP after CA exist.^14^ A narrative review identified two clinical trials randomising patients to lower (65–75 mmHg) and higher (80–100 mmHg) MAP targets.^5^, ^6^, ^15^ However, that review did not include a systematic search, meta‐analyses or trial sequential analyses (TSA).^15^ A systematic review from 2015 identified only nine observational studies investigating the relationship between blood pressure and neurologic outcomes.^16^ This systematic review is important to explore whether targeting a higher compared to a lower MAP after CA is beneficial, harmful or uncertain for CA patients, to highlight knowledge gaps and to demonstrate ambiguity or a signal of benefit for a higher MAP currently and when new RCTs are published.

This protocol for a systematic review and individual patient meta‐analysis aims to compare the effects of targeting a higher or lower MAP in CA patients. We will restrict this review to CA patients, since the haemodynamic severity and pathophysiology of global hypoxic brain injury may differ greatly from other brain injury types.^17^

## 2 METHODS

This protocol is based on the Preferred Reporting Items for Systematic Reviews and Meta‐Analysis Protocols (PRISMA‐P) guidelines for reporting systematic reviews of healthcare interventions.^18^, ^19^

### 2.1 Randomised trials informing choice of collected data

At least three randomised clinical trials (RCTs) conducted on different MAP targets after out‐of‐hospital cardiac arrest (OHCA) exist.^5^, ^6^, ^13^ Their methodology, data and outcomes will inform a priori this systematic review.

### 2.2 Eligibility

- RCTs irrespective of design, setting, blinding, publication status, publication year and reported outcomes.
- Studies with at least the abstract available in English.
- Patients ≥ 18 years treated after return of spontaneous circulation (ROSC) in an ambulance, emergency department and/or ICU irrespective of sex and comorbidities. If trials only include a subset of eligible participants, they will be included if (1) separate data on the eligible participants are available or (2) more than 90% are eligible.
- Experimental intervention: MAP targets ≥71 mmHg.
- Control intervention: standard MAP target of 65–70 mmHg or lower.
- Any type of co‐intervention which is intended to be delivered similarly to the experimental and control groups.

### 2.3 Outcome measures

#### 2.3.1 Primary outcomes

- All‐cause 90‐ and 180‐day mortality.
- Functional outcome defined by the cerebral performance category (CPC) scale or the modified Rankin scale (mRS), dichotomised into favourable and unfavourable outcomes (CPC 1–2 vs. CPC 3–5 or mRS 0–3 vs. 4–6).^20^

We will in all primary analyses include survival status and functional outcome reported at a time point closest to 180 days after randomisation. If outcome results are only reported at hospital discharge then these data will be included in the analysis.

#### 2.3.2 Secondary outcomes

- ICU mortality (trialist defined).
- Health‐related quality of life (any validated continuous scale).
- New arrhythmia resulting in haemodynamic compromise (trialist defined).
- Hospital‐free days within 30 days.
- Serious adverse events: any untoward medical occurrence that results in death, is life‐threatening, requires or prolongs hospitalisation or results in persistent or significant disability. In many trials, we expect very heterogeneous serious adverse events reporting that does not adhere to the International Council for Harmonisation of Technical Requirements for Registration of Pharmaceuticals for Human Use – Good Clinical Practice (ICH‐GCP).^21^ We will include serious adverse events defined as such by the trialists or report the proportion of participants with events that we consider fulfil the ICH‐GCP definition. If studies report several such events, we will choose the highest proportion reported in each trial. We will analyse each component separately.

#### 2.3.3 Exploratory outcomes

- Acute kidney injury defined according to the Kidney Disease Improving Global Outcome (KDIGO) criteria 0–3.^22^
- Cardiac functional capacity, as defined by the New York Heart Association classification 1–4.^23^
- Cardiac function at 90–180 days (latest available) defined by an ejection fraction < 40%.
- Levels of the brain injury biomarkers neuron‐specific enolase (NSE) and neurofilament light (NfL) measured at 48 h from the cardiac arrest.^24^, ^25^
- Level of high sensitivity troponin (hsTNT), a cardiac injury biomarker, at 12, 24, 48 and 72 h during ICU care (either from cardiac arrest, ICU admission or randomisation based on the individual trial's strategy used) and a calculation of the area under the hsTNT curve.
- Hospital mortality (trialist defined).
- Time to extubation.
- New CA in the ICU (trialist defined).
- Severe hypoxia (partial pressure of oxygen PaO_2_ < 8 kPa).

### 2.4 Search strategy

#### 2.4.1 Databases

- Cochrane Central Register of Controlled Trials (CENTRAL);
- MEDLINE (Ovid, 1946 onwards);
- Embase (Ovid, 1980 onwards);
- LILACS (Bireme, 1982 onwards);
- BIOSIS (Thomson Reuters, 1926 onwards);
- CINAHL (EBSCO Publishing, 1961 onwards);
- Scopus (Elsevier, 1788 onwards);
- Web of Science Core Collection (Clarivate, 1900 onwards).

A preliminary search strategy developed for MEDLINE (Ovid) is included in Supplementary Online Material. We will adapt this search strategy for MEDLINE (Ovid) and the other databases.

#### 2.4.2 Other resources

We will hand‐search the reference lists of the included randomised clinical trials, systematic reviews and other types of reviews to find unidentified RCTs and, if indicated, email the authors to retrieve further information. We will also search for unidentified randomised trials on:

- ClinicalTrials.gov (https://clinicaltrials.gov);
- The World Health Organisation International Clinical Trials Registry (http://apps.who.int/trialsearch/);
- Google Scholar (https://scholar.google.com/); and
- The Turning Research into Practice (TRIP) database (https://www.tripdatabase.com/).

In addition, we will include unpublished trials and trials found in the grey literature.

### 2.5 Study selection

The search results will be uploaded to Covidence (Veritas Health Innovation, Melbourne, Australia). Two authors (MS and VN) will screen the full text of all the retrieved studies to identify trials for inclusion. The studies will be coded initially as No/Maybe/Yes, and the reasons for excluding ineligible studies will be documented. We will use Cohen's Kappa Coefficient as a measure of agreement. In case of disagreement, a third author will provide an additional review, and to avoid potential intellectual scientific conflicts of interest (authors scoring their own study), we will involve independent (non‐conflicted) authors (JCJ and NN). We will identify and exclude duplicate and multiple reports of the same trial, so that each trial, rather than each report, is the unit of interest in the review. The study selection process will be presented in a PRISMA flow diagram.

### 2.6 Data extraction and management

The data will be extracted from the included trials and validated by a pair of two authors (MS, VN, KA, AA). Any authors involved in any included trial will not extract data from or assess the risk of bias in those trials. Any disagreement concerning the extracted data will be resolved by discussion with a third author (NN or JCJ). Duplicate publications and companion papers will be identified to evaluate the trials and all available data simultaneously to maximise data extraction and correct bias assessment. If indicated, we will email the trial authors to request data that may not have been sufficiently included in the primary publication. The following trial data will be extracted:

- Trial characteristics: bias risk components, trial design (parallel, factorial or crossover), period and number of sites, countries where the trial was conducted, number of intervention arms, length of follow‐up and inclusion and exclusion criteria.
- Participant characteristics and comorbidities: number of randomised participants, analysed participants, participants lost to follow‐up, mean age, age range, sex ratio, specific patient‐based inclusion criteria, CA and treatment characteristics (e.g., presence of chronic hypertension or not, shockable or non‐shockable rhythm, TTM or no TTM).
- Experimental intervention characteristics: MAP target, vasopressor use and type or intravenous fluid type and dose.
- Control intervention characteristics: MAP target, vasopressor used, type of fluid management strategy.
- Co‐intervention characteristics: type, dose and duration of co‐intervention and administration mode.
- Outcomes: primary and secondary outcomes specified and collected, time points reported and differences in planned and reported outcomes.
- Adverse effects, new onset cardiac arrhythmia, new CA, duration of mechanical ventilation, prevalence of severe hypoxia.

### 2.7 Individual data requested from studies

The study investigators will be contacted so that we can include individual patient data on the following:

1. Treatment group assignment
2. Patient age
3. Gender
4. Arrest location: out of hospital or in hospital
5. Chronic hypertension as comorbidity (based on antihypertensive medication prescriptions)
6. Initial rhythm: shockable or non‐shockable
7. Presumed cause of arrest: cardiac or non‐cardiac
8. Time to ROSC
9. ST‐elevation myocardial infarction: yes/no
10. TTM target: 33°C, 36°C, 37.5°C or no TTM
11. Highest used vasopressor and inotrope dose during the intervention (noradrenaline, adrenaline, phenylephrine, dobutamine, dopamine and vasopressin)
12. Highest used total vasopressor load during the intervention^26^
13. New CA in the ICU
14. New arrhythmia resulting in haemodynamic compromise
15. Severe hypoxia despite treatment (PaO_2_ less than 8 kPa)
16. NSE and NfL levels measured 48 h from the arrest
17. Time to extubation in hours
18. ICU length of stay
19. Death in the ICU
20. Death in the hospital
21. Mortality at longest follow‐up point
22. Functional outcome defined as CPC 1–5 and/or mRS 0–5
23. Favourable functional outcome defined as CPC 1 or 2 or mRS 0–3 at longest follow‐up time

### 2.8 Assessment of risk of bias

The bias assessments will be based on the Cochrane Risk of Bias tool, version 2 (Cochrane Denmark).^27^ We include the methodology used to assess bias as a supplement.

### 2.9 Differences between the protocol and the review

Any deviations from this protocol will be reported in a dedicated section.

### 2.10 Statistical analysis

We will use R (R core team, Vienna, Austria) and/or Stata (StataCorp LLC, Texas) for all analyses.

Individual patient data meta‐analysis.

Analyses of the individual‐level patient data from all the randomised participants will be conducted. Baseline comparisons indexed by treatment group will be performed using Fisher's exact test, Student's *t*‐test for the normally distributed data and the Mann–Whitney U test for the non‐parametric data. These comparisons will be used to explore any differences between the baseline characteristics and will be interpreted exploratorily. The results will be reported as numbers (%), standardised mean differences (SMD) or medians (interquartile range, IQR). The outcomes will be analysed on an intention‐to‐treat basis. We will assess the effects of a total of seven primary and secondary outcomes and consider a two‐sided *p* value <.013 as the threshold for statistical significance.^28^ Dichotomous outcomes in the two treatment groups will be compared using multivariable logistic regression modelling, adjusting for site as a random effect and reported as relative risks. We will calculate 95% confidence intervals (CI) for relative risks using either the nlcom‐command in Stata or G‐computation in R. The numbers at risk in the two intervention groups and the numbers and proportions of events will be reported. Continuous outcomes will be analysed using linear regression adjusted for the baseline value of the dependent variable (if available) and trial site as a random intercept.

### 2.11 Handling missing data

Missing data will be handled according to Jakobsen et al.’s recommendations.^29^ We anticipate that the proportion of missing values for the primary and secondary outcomes will be less than 5%. However, in a secondary analysis, we will consider using multiple imputation and/or present best‐worst and worst‐best case scenarios if it is not valid to ignore missing data.^29^

### 2.12 Underlying statistical assumptions

We will systematically assess the underlying statistical assumptions for all the statistical analyses.^30^, ^31^ For all primary and secondary regression analyses, we will test for major interactions between the site and the intervention variable and whether it shows a clinically important effect. If the interaction is significant, we will consider presenting a separate analysis for each trial or site and an overall analysis including the interaction term in the model.^30^, ^31^

### 2.13 Underlying statistical assumptions for dichotomous outcomes

We will assess whether the deviance divided by the degrees of freedom is significantly larger than 1 to assess for relevant overdispersion, which is the presence of greater variability (statistical dispersion) in a dataset than would be expected based on a given statistical model. In that case, we will consider using a maximum likelihood estimate of the dispersion parameter. After checking if the number of participants is larger than 10 (rule of thumb) per site, we will consider pooling the data from the small sites if the number of participants is too low.^31^

### 2.14 Trial sequential analysis

Cumulative meta‐analyses may produce random errors because of sparse data and multiple testing of the accumulated data. The TSA program can mitigate such risks (Copenhagen Trial Unit; http://www.ctu.dk/tsa/).^32^ The TSA estimates the diversity‐adjusted required information size (DARIS) (i.e., the number of participants needed in a meta‐analysis to detect or reject certain intervention effects), minimising the risk of random errors. TSA provides the anticipated intervention effect, the variance of the anticipated difference in the intervention effect and the acceptable risk of a falsely rejected null hypothesis (alpha). It also provides the acceptable risk of falsely failing to reject a null hypothesis (beta) and the variance of the intervention effect estimates between the included trials. We will search for suitable empirical data to determine and predefine the anticipated intervention effects. If no suitable data are found, a pragmatic estimation of the anticipated intervention effects is as follows:

- Regarding the analysis of dichotomous outcomes, an intervention effect equal to at least a risk ratio reduction of 25%, an alpha of 5% and 90% power will be anticipated.

Given the use of TSA analysis, significance testing can be conducted each time a new trial is included in the meta‐analysis. Using the DARIS, trial sequential monitoring boundaries will be constructed, enabling the determination of statistical inferences concerning the cumulative meta‐analyses that have not been met with the DARIS. Firm evidence for benefit or harm may be established only if a given trial's sequential monitoring boundary (upper boundary of benefit, lower boundary of harm or area of futility) is crossed before reaching the DARIS, in which case further trials may be superfluous. Conversely, if a boundary is not surpassed, it may be necessary to continue with further trials before a certain intervention effect can be firmly detected or rejected. Firm evidence for the lack of a postulated intervention effect can also be assessed using TSA. This is the case when the cumulative *z*‐score crosses the trial sequential boundaries for futility. TSA‐adjusted CIs will be reported in addition to the unadjusted naive 95% CIs, as they are adjusted for the lack of information, thus providing a better estimation of the true CI. If TSA is impossible because information is lacking, more lenient analysis that increases the anticipated intervention effect (in these cases, the TSA‐adjusted CI is overly optimistic) will be performed. For dichotomous outcomes, estimation of the DARIS based on an anticipated intervention effect will be performed as follows: where the observed proportion of participants with an outcome in the control group has an alpha of 2.0% for our primary and secondary outcomes and 5.0% for our exploratory outcomes, a beta of 10% and a diversity as suggested by the trials in the meta‐analysis. Regarding continuous outcomes, an estimation of the DARIS will be based on a minimal clinically important difference of SD/2, the SD observed in the control group, an alpha of 2.0% for our primary and secondary outcomes and 5.0% for our exploratory outcomes, a beta of 10% and a diversity as suggested by the trials in the meta‐analysis. Potentially difficult decisions will be documented, and their impact on the findings will be assessed using sensitivity analyses.

### 2.15 Meta‐analysis of aggregate data

A meta‐analysis will be performed as a supplementary analysis according to the recommendations in the Cochrane Handbook for Systematic Reviews of Interventions.^33^ The assessment of intervention effects will be performed with random‐effects and fixed‐effects meta‐analyses; the result with the highest *p* value will be reported as the primary result and the less conservative results as a sensitivity analysis.^28^ In case of a substantial discrepancy between the two methods' results, both will be reported and discussed.

### 2.16 Assessment of heterogeneity

Forest plots will be constructed to visualise and assess any possible signs of heterogeneity. Statistical heterogeneity will be assessed using the chi‐square test (threshold *p* < .10), the quantities of heterogeneity will be measured with the *I* ^2^ statistic, and possible heterogeneity will be assessed with relevant subgroup analyses. Ultimately, the meta‐analysis may not proceed, given significant heterogeneity.

### 2.17 Subgroup analysis and investigation of heterogeneity

We will perform the following tests of interaction and subgroup analyses on all outcomes:

- Comparison of ages <65 or ≥65 years based on subgroups from a large OHCA RCT^34^;
- Comparison of effects based on patient history of chronic hypertension;
- Comparison of effects between patients with a shockable or a non‐shockable initial rhythm;
- Comparison based on time to ROSC <25 or ≥25 min based on subgroups from a large OHCA RCT^34^;
- Comparison based on circulatory shock on first assessment based on the presence of a cardiac Sequential Organ Failure Assessment (SOFA) score of 4 on randomisation (dopamine > 15 μg/kg/min OR epinephrine > 0.1 μg/kg/min OR norepinephrine > 0.1 μg/kg/min)^35^;
- Comparison of effects between patients treated without TTM and those treated with TTM targeting 33°C.

### 2.18 Statistical reports

The data on all outcomes will be analysed by two independent individuals (MS and VN).^31^ Two independent statistical reports will be sent to the principal investigator and shared with the steering group and the author group. If there are discrepancies between the two primary statistical reports, possible reasons will be identified, and the steering group will decide which is the most correct result. A final statistical report will be prepared, and all three statistical reports will be published as Supplementary Material.^31^

### 2.19 Summary of findings

The GRADE system will be used to assess the certainty of the body of evidence associated with each outcome, and we will construct a summary of findings (SoF) table using GRADEpro software (McMasters University).^33^ The GRADE approach appraises the certainty of the body of evidence based on the extent to which one can be confident that an estimate of effect or association reflects the item being assessed. We will assess the GRADE levels of evidence as high, moderate, low or very low and downgrade the evidence by one or two levels depending on the following certainty measures: the within‐study risk of bias, directness of the evidence, heterogeneity of the data, precision of the effect estimates and risk of publication bias. We will use TSA to assess the imprecision of the effect estimates. We will use the methods and recommendations described in Chapter 8 (Section 8.5) and Chapter 1272 of the Cochrane Handbook for Systematic Reviews of Interventions.^33^ We will justify all decisions to downgrade the certainty of the studies in footnotes, adding comments where necessary. We will include all trials in our analyses and conduct a sensitivity analysis, excluding trials with a high risk of bias. If the results are similar, we will base our SoF table and conclusions on the overall analysis. If they differ, we will base our SoF table and conclusions on the trials with a low risk of bias.

### 2.20 Patient and public involvement

We developed this protocol for a systematic review without patient and public involvement.

### 2.21 Repeating the analysis given more studies on this topic

Larger studies are underway to examine different MAP targets after OHCA.^36^ In addition, a large 2 × 2 × 2 factorial RCT is being planned (Nielsen, personal communication, March 2022) that will include standard and high MAP as two intervention groups following OHCA of all aetiologies. This protocol will be used to include data from these and other trials expected to report in the next few years.

### 2.22 Ethics and dissemination

As the individual patient data meta‐analysis may include identifiable data, formal approval or review of an ethical committee may be required for this systematic review in accordance with local protocols. The results of this systematic review will be disseminated through publication in a leading peer‐reviewed journal.

## 3 DISCUSSION

Current post‐CA care guidelines highlight the need for further studies on optimal MAP targets after CA.^14^ This protocol aims to assess the effects of targeting higher or lower MAP targets in patients after CA and the possible effects on mortality, functional outcome and occurrence of adverse events. This systematic review will provide data on the recommended MAP after CA patients and inform future trials.

The aetiology of CA varies, but management recommendations concur.^1^ We will include presumed CA aetiology and initial cardiac rhythm as subgroup analyses to determine whether these suggest different effects of a higher MAP target. As studies have suggested a higher likelihood of the optimal MAP being greater than the recommended 65–70 mmHg in those with chronic hypertension,^37^, ^38^ we will include the presence of chronic hypertension as a subgroup. Since the use of targeted temperature management (TTM) targeting lower temperatures is associated with a higher prevalence of hypotension,^39^ we will perform a subgroup analysis of patients treated with different TTM strategies.

This protocol has several strengths, including use of a predefined methodology based on the PRISMA‐P guidelines and the GRADE system, TSA and the five‐step assessment by Jakobsen et al. for better validation of meta‐analytical results in systematic reviews.^18^, ^28^, ^32^, ^33^ This protocol considers the risks of both random errors and systematic errors. Individual patient data meta‐analysis will enable us to report adjusted outcomes and assess outcomes in certain relevant subgroups.

Our protocol has limitations. The primary limitation is that we will include various types of therapies used to achieve the targeted MAP. The effect of increasing MAP may depend on the intervention used, in terms of efficacy and adverse effects. Another limitation is that the effect of the intervention on the outcome will depend on the patients' comorbidities, factors present at resuscitation, the aetiology of the arrest and concomitant ICU treatment, such as the TTM target temperature. To minimise these limitations, we plan to carefully assess clinical and statistical heterogeneity, including several subgroup analyses, but these subgroup analyses will presumably be underpowered. Another limitation is the large number of comparisons, which increases the risk of family‐wise errors. To minimise this limitation, we have adjusted our thresholds for significance according to the total number of primary and secondary outcomes. Nevertheless, we have not adjusted our thresholds for significance according to the large number of subgroup analyses. The substantial risks of type I errors will also be considered when interpreting our results.

## 4 CONCLUSION

The optimal MAP after cardiac arrest is currently unknown. This planed IPDMA will inform clinicians about whether a higher MAP than currently recommended by guidelines could improve patient outcome. Repeating this IPDMA after new studies have been published will allow for more certainty about any possible treatment effect also including relevant sub‐groups of patients.

## AUTHOR CONTRIBUTIONS

Markus B. Skrifvars, Matti Reinikainen, Johanna Hästbacka, Marjaana Tiainen, Niklas Nielsen and Janus C. Jakobsen planned the study. Koen Ameloot, Johannes Grand, Christian Hassager and Jesper Kjaergard have taken part in the original studies included in this systematic review and patient data meta‐analysis. Markus Harboe Olsen, Josef Dankiewicz and Caroline Kamp Jørgensen contributed to the statistical analysis plan. All authors took part in writing the manuscript and the final study plan. All authors have read and approved the final version.

## FUNDING INFORMATION

This study has been funded by Sigrid Juselius Stifelse (grant number 8050) and the Academy of Finland (grant number 341277).

## CONFLICT OF INTEREST

Markus B. Skrifvars reports speakers fees from BARD Medical (Ireland). All other authors report no competing interests.

## Supporting information


**Appendix S1** Supporting Information

## Data Availability

All data without individual patient information will be shared freely. The sharing of pseudoanonymised patient data will be discussed and decided by the investigators of each included trial and the legal recommendations of each trial.
