# Supplementary material for: Protocol for an individual patient data meta‐analysis on blood pressure targets after cardiac arrest
Source: Acta Anaesthesiol Scand. 2022 Jun 9;66(7):890–7. doi: 10.1111/aas.14090 (PMC9543739; doi:10.1111/aas.14090)
Supplement: Supplementary file 1 — Appendix S1 Supporting Information [file AAS-66-890-s001.pdf]

Supplementary online material:

Higher compared to lower mean arterial blood pressure in patients resuscitated from cardiac arrest: protocol for a systematic reviews with individual patient data meta-analysis and trial sequential analysis

Markus B Skrifvars[1], Koen Ameloot[2–4], Johannes Grand[5], Matti Reinikainen[6], Johanna Hästbacka[7], Ville Niemelä[7], Christian Hassager[6], Jesper Kjaergard[6], Anders Åneman[8], Marjaana Tiainen[9], Niklas Nielsen[10], Josef Dankiewicz[11], Markus Harboe Olsen[12,13], Caroline Kamp Jørgensen[12], Janus C Jakobsen[12, 14]

[1]Department of Emergency Care and Services, Helsinki University Hospital and University of Helsinki, Helsinki, Finland
[2]Department of Cardiology, Ziekenhuis Oost-Limburg, Genk, Belgium
[3]Department of Cardiology, University Hospitals Leuven, Leuven, Belgium
[4]Faculty of Medicine and Life Sciences, University Hasselt, Diepenbeek, Belgium
[5]Department of Cardiology, Copenhagen University Hospital – Rigshospitalet, Copenhagen, Denmark
[6]Department of Intensive Care, Kuopio University Hospital and University of Eastern Finland, Kuopio, Finland
[5]Department of Cardiology, Copenhagen University Hospital – Rigshospitalet, Copenhagen, Denmark
[7]Department of Anesthesiology, Intensive Care, and Pain Medicine, Helsinki University and Helsinki University Hospital, Helsinki, Finland
[8]Intensive Care Unit, Liverpool Hospital, South Western Sydney Local Health District; University of New South Wales; Faculty of Medicine and Health Sciences, Macquarie University, Sydney, Australia
[9]Department of Neurology, Helsinki University Hospital and University of Helsinki, Helsinki, Finland
[10]Lund University and Department of Clinical Sciences Lund, Anaesthesia and Intensive Care, Helsingborg Hospital, Lund, Sweden
[11]Department of Clinical Sciences Lund, Sections of Cardiology, Skåne University Hospital Lund, Lund University and Clinical Studies Sweden
[12] Copenhagen Trial Unit, Centre for Clinical Intervention Research, Copenhagen University Hospital − Rigshospitalet, The Capital Region, Copenhagen, Denmark.
[13] Department of Neuroanaesthesiology, The Neuroscience Centre, Copenhagen University Hospital − Rigshospitalet, Denmark.
[14] Department of Regional Health Research, Faculty of Health Sciences, University of Southern Denmark, Odense, Denmark

Address for correspondence: Markus Skrifvars, Meilahti Hospital (MEM2B), Haartmaninkatu 9, 000290 HUS, Helsinki, Finland. Email: markus.skrifvars@hus.fi, Phone:+358405137862

1. Bias assessment

The methodology used in the included trials will be evaluated according to the following bias domains: bias arising from the randomisation process, bias due to deviation from intended interventions, bias due to missing outcome data, bias in measurement of outcomes, bias arising from selective reporting of results and overall risk of bias.

<u>Bias arising from the randomisation process</u>

This includes the allocation sequence generation and concealment and possible baseline differences between the trial arms.

Low risk of bias

The group allocations are adequately concealed, AND baseline imbalances between the intervention groups at baseline seem compatible with chance, AND an adequate (random or otherwise unpredictable) method was used to generate allocation sequence, OR no information about the method used to generate the allocation sequence was reported.

Some concerns

There was adequate allocation concealment, AND there appeared to be problems with the method of sequence generation, OR there were problems with baseline imbalances suggesting problems with the randomisation process, OR no information was provided about the allocation concealment, AND baseline imbalances between the intervention groups appear compatible with chance OR there is not enough information to answer the signalling questions.

High risk of bias

The allocation sequence was not concealed, OR information was not provided about the concealment of the allocation sequence AND baseline imbalances suggest a problem with the randomisation process.

<u>Bias due to deviation from intended interventions</u>

Low risk of bias

The treatment providers, patients and study personnel were not aware of the study intervention groups during the trial, OR the participants, treating providers and study personnel were not aware of the intervention groups during the trial, but possible deviations from the intended interventions likely reflected usual practice, OR the participants, treating providers or study personnel were aware of the intervention groups during the trial, but possible deviations from the intended interventions were not likely to impact study outcomes AND no participants were analysed in the wrong intervention groups (that is, on the basis of the intervention received instead of what was mandated by the randomised allocation).

Some concerns

The participants, treatment providers or study personnel were aware of the intervention groups, and there is limited information on whether there were deviations from current practice that may have impacted outcomes, AND there were imbalances between the intervention groups OR some participants were analysed in the wrong intervention groups (on the basis of the intervention received rather than what was mandated by the randomised allocation), but there was minimal potential for a substantial impact on the estimated effect of the intervention.

High risk of bias

The participants, carers or study personnel were aware of the intervention groups, and deviations were evident from the intended interventions resulting in imbalances between the intervention groups that likely affected the outcomes, OR some participants were analysed in the wrong intervention groups (on the basis of the intervention received rather than mandated by the randomised allocation) AND here was potential for substantial impacts on the estimated effects of the intervention.

<u>Bias due to missing outcome data</u>

Low risk of bias

No missing data OR non-differential missing data (similar proportions of and comparable reasons for missing data in the intervention groups) OR evidence of robustness of effect estimates for missing data (based on appropriate statistical methods for handling missing data, including sensitivity analysis).

Some concerns

An unclear degree of missing data or uncertainty about the information on the proportion and reasons for the missing data in the compared groups AND no evidence that the effect estimate is robust despite missing data.

High risk of bias

A high degree of missing data AND differential missing data (different proportions of or reasons for missing data in the intervention groups) AND there is no such evidence that effect estimates are robust for missing data.

<u>Bias in measurement of outcomes</u>

Low risk of bias

The outcome assessors were not aware of the intervention received by the study participants OR the outcome assessors were aware of the intervention received by the study participants, but the assessment of outcome was likely not influenced by knowledge of the intervention received.

Some concerns

No information is available to determine whether assessments of outcomes are likely to have been influenced by knowledge of the intervention received.

High risk of bias

The assessment of outcomes was likely influenced by knowledge about the intervention received by the study participants.

<u>Bias arising from selective reporting of results</u>

Low risk of bias

Reported outcome data are not likely to have been selected, based on the results, from multiple outcome measurements (e.g. scales, definitions, time points) within outcome domains, and reported outcomes are unlikely to have been selected, based on the results, from multiple analyses of the data.

Some concerns

Not enough information is available to exclude the possibility that reported outcomes were selected, based on results, from multiple outcome measures (e.g. scales, definitions,

time points) within the outcome domain or from multiple analyses of the data. Given that analysis intentions are often unavailable or reported in insufficient detail, we think this will be the default judgement for most trials.

High risk of bias

Reported outcome data are likely to have been selected, based on results, from multiple outcome measurements (e.g. scales, definitions, time points) within the outcome domain or from multiple analyses of the data (or both).

<u>Overall assessment of risk of bias</u>

Low risk of bias

The study is judged to be at low risk of bias in all domains.

High risk of bias

The study is judged to be at high risk of bias or to exhibit concerns in at least one domain. In a subgroup analysis, we will compare the intervention effects of trials judged to be at low risk of bias with trials judged to be at high risk of bias. An assessment of the domains of missing outcome data, risk of bias in measurement of the outcome and risk of bias in selection of the reported result for each outcome result will be undertaken. Accordingly, an assessment of the bias risk for each outcome will be performed in each trial. The primary conclusions will be

based on the results of the primary outcome results regarding the overall low risk of bias. Both the primary and secondary conclusions will be presented using summary tables.

2. Preliminary search strategy

Search strategies for MAP for CA (JC Jakobsen and M Skrifvars)

Preliminary searches prepared 23 March 2022

Cochrane Central Register of Controlled Trials (Latest issue) in the Cochrane Library (*3404 hits*)
#1 MeSH descriptor: [Arterial Pressure] explode all trees
#2 (((arterial or intraarterial or blood or pulse) and (pressure or tension)) or MAP)
#3 #1 or #2
#4 (vasopress* or inotrop* or intraveneous fluid* or noradrenaline or norepinephrine or dopamine or dobutamine or metoprolol or levophed or seloken or iloprost)
#5 #3 or #4
#6 MeSH descriptor: [Heart Arrest] explode all trees
#7 MeSH descriptor: [Resuscitation] explode all trees
#8 (((cardiac or heart) and arrest) or resuscitat* or CPR or OHCA or CA)
#9 #6 or #7 or #8
#10 #5 and #9
#11 MeSH descriptor: [Adult] explode all trees
#12 MeSH descriptor: [Adolescent] explode all trees
#13 #10 and (#11 or #12)

MEDLINE Ovid (1946 to the date of the search) (*2319 hits*)
1. exp Arterial Pressure/
2. (((arterial or intraarterial or blood or pulse) and (pressure or tension)) or MAP).mp. [mp=title, abstract, original title, name of substance word, subject heading word, floating sub-heading word, keyword heading word, organism supplementary concept word, protocol supplementary concept word, rare disease supplementary concept word, unique identifier, synonyms]
3. 1 or 2
4. (vasopress* or inotrop* or intraveneous fluid* or noradrenaline or norepinephrine or dopamine or dobutamine or metoprolol or levophed or seloken or iloprost).mp. [mp=title, abstract, original title, name of substance word, subject heading word, floating sub-heading word, keyword heading word, organism supplementary concept word, protocol supplementary concept word, rare disease supplementary concept word, unique identifier, synonyms]
5. 3 or 4
6. exp Heart Arrest/
7. exp Resuscitation/
8. (((cardiac or heart) and arrest) or resuscitat* or CPR or OHCA or CA).mp. [mp=title, abstract, original title, name of substance word, subject heading word, floating sub-heading word, keyword heading word, organism supplementary concept word, protocol supplementary concept word, rare disease supplementary concept word, unique identifier, synonyms]
9. 6 or 7 or 8
10. 5 and 9
11. limit 10 to ("adolescent (13 to 18 years)" or "young adult (19 to 24 years)" or "adult (19 to 44 years)" or "young adult and adult (19-24 and 19-44)" or "middle age (45 to 64 years)" or "middle aged (45 plus years)" or "all aged (65 and over)" or "aged (80 and over)")
12. (randomized controlled trial or controlled clinical trial or retracted publication or retraction of publication).pt. or clinical trials as topic.sh. or trial.ti.

13. (random* or blind* or placebo* or meta-analys*).mp. [mp=title, abstract, original title, name of substance word, subject heading word, floating sub-heading word, keyword heading word, organism supplementary concept word, protocol supplementary concept word, rare disease supplementary concept word, unique identifier, synonyms]
14. 11 and (12 or 13)

Embase Ovid (1974 to the date of the search) (*3000 hits*)
1. exp arterial pressure/
2. (((arterial or intraarterial or blood or pulse) and (pressure or tension)) or MAP).mp. [mp=title, abstract, heading word, drug trade name, original title, device manufacturer, drug manufacturer, device trade name, keyword heading word, floating subheading word, candidate term word]
3. 1 or 2
4. (vasopress* or inotrop* or intraveneous fluid* or noradrenaline or norepinephrine or dopamine or dobutamine or metoprolol or levophed or seloken or iloprost).mp. [mp=title, abstract, heading word, drug trade name, original title, device manufacturer, drug manufacturer, device trade name, keyword heading word, floating subheading word, candidate term word]
5. 3 or 4
6. exp heart arrest/
7. exp resuscitation/
8. (((cardiac or heart) and arrest) or resuscitat* or CPR or OHCA or CA).mp. [mp=title, abstract, heading word, drug trade name, original title, device manufacturer, drug manufacturer, device trade name, keyword heading word, floating subheading word, candidate term word]
9. 6 or 7 or 8
10. 5 and 9
11. limit 10 to (adult <18 to 64 years> or aged <65+ years>)
12. Randomized controlled trial/ or Controlled clinical trial/ or retracted article/ or (erratum or tombstone).pt. or trial.ti. or yes.nr.
13. (random* or blind* or placebo* or meta-analys*).mp. [mp=title, abstract, heading word, drug trade name, original title, device manufacturer, drug manufacturer, device trade name, keyword heading word, floating subheading word, candidate term word]
14. 11 and (12 or 13)

LILACS (Bireme; 1982 to the date of the search) (*633 hits)*
(((arterial or intraarterial or blood or pulse) and (pressure or tension)) or MAP) or (vasopress$ or inotrop$ or intraveneous fluid$ or noradrenaline or norepinephrine or dopamine or dobutamine or metoprolol or levophed or seloken or iloprost) [Words] and (((cardiac or heart) and arrest) or resuscitat$ or CPR or OHCA or CA) [Words]

CINAHL (Ebsco host; the date of the search will be given at the review stage) (*1354 hits*)
S14 S12 AND S13
S13 MH adult OR MH adolescence
S12 S10 AND S11
S11 TX (random* or blind* or placebo* or meta-analys*)
S10 S5 AND S9
S9 S6 OR S7 OR S8
S8 TX (((cardiac or heart) and arrest) or resuscitat* or CPR or OHCA or CA)
S7 MH Resuscitation

S6 MH Heart Arrest
S5 S3 OR S4
S4 TX (vasopress* or inotrop* or intraveneous fluid* or noradrenaline or norepinephrine or dopamine or dobutamine or metoprolol or levophed or seloken or iloprost)
S3 S1 OR S2
S2 TX (((arterial or intraarterial or blood or pulse) and (pressure or tension)) or MAP)
S1 MH Arterial Pressure

BIOSIS (Web of Science; 1969 to the date of the search) (*2316 hits*)
#7 #5 AND #6
#6 TI=(random* or blind* or placebo* or meta-analys* or trial*) OR TS=(random* or blind* or placebo* or meta-analys*)
#5 #3 AND #4
#4 TS=(((cardiac or heart) and arrest) or resuscitat* or CPR or OHCA or CA)
#3 #2 OR #1
#2 TS=(vasopress* or inotrop* or intraveneous fluid* or noradrenaline or norepinephrine or dopamine or dobutamine or metoprolol or levophed or seloken or iloprost)
#1 TS=(((arterial or intraarterial or blood or pulse) and (pressure or tension)) or MAP)

Science Citation Index Expanded (1900 to the date of the search) and Conference Proceedings Citation Index – Science (1990 to the date of the search) (Web of Science) (*4391 hits*)
#7 #5 AND #6
#6 TI=(random* or blind* or placebo* or meta-analys* or trial*) OR TS=(random* or blind* or placebo* or meta-analys*)
#5 #3 AND #4
#4 TS=(((cardiac or heart) and arrest) or resuscitat* or CPR or OHCA or CA)
#3 #2 OR #1
#2 TS=(vasopress* or inotrop* or intraveneous fluid* or noradrenaline or norepinephrine or dopamine or dobutamine or metoprolol or levophed or seloken or iloprost)
#1 TS= (((arterial or intraarterial or blood or pulse) and (pressure or tension)) or MAP)

Scopus (the date of the search will be given at review stage) (*2049 hits*)
#9 #7 AND #8
#8 TITLE-ABS-KEY (adult* OR adolescent*)
#7 #5 AND #6
#6 TITLE-ABS-KEY (random* OR blind* OR placebo* OR meta-analys*)
#5 #3 AND #4
#4 TITLE-ABS-KEY (((cardiac OR heart) AND arrest) OR resuscitat* OR cpr OR ohca OR ca)
#3 #1 OR #2
#2 TITLE-ABS-KEY (vasopress* OR inotrop* OR intraveneous AND fluid* OR noradrenaline OR norepinephrine OR dopamine OR dobutamine OR metoprolol OR levophed OR seloken OR iloprost)
#1 TITLE-ABS-KEY (((arterial OR intraarterial OR blood OR pulse) AND (pressure OR tension)) OR map)
